# Label-Free Detection of Cellular Aβ Accumulation and Mitochondrial Dysfunction in AD Cell Models via Raman Microscopy

**DOI:** 10.1021/acs.analchem.5c06200

**Published:** 2026-06-23

**Authors:** Yusuke Mitsuoka, Takeshi Morimoto, Kazuki Bando, Susumu Hara, Katsumasa Fujita, Kohji Nishida

**Affiliations:** † Department of Ophthalmology, 13013 The University of Osaka Graduate School of Medicine, Suita, Osaka 565-0871, Japan; ‡ Department of Advanced Visual Neuroscience, The University of Osaka Graduate School of Medicine, Suita, Osaka 565-0871, Japan; § Department of Applied Physics, Osaka University, Suita, Osaka 565-0871, Japan; ∥ Advanced Photonics and Biosensing Open Innovation Laboratory, National Institute of Advanced Industrial Science and Technology (AIST), Suita, Osaka 565-0871, Japan; ⊥ Premium Research Institute for Human Metaverse Medicine (WPI-PRIMe), The University of Osaka, Suita, Osaka 565-0871, Japan; # Transdimensional Life Imaging Division, Institute for Open and Transdisciplinary Research Initiatives, The University of Osaka, Suita, Osaka 565-0871, Japan; ¶ Institute for Open and Transdisciplinary Research Initiatives, Osaka University, Suita, Osaka 565-0871, Japan; ∇ Integrated Frontier Research for Medical Science Division, Institute for Open and Transdisciplinary Research Initiatives, Osaka University, Suita, Osaka 565-0871, Japan

## Abstract

Alzheimer’s disease (AD) is a neurocognitive disorder characterized by the accumulation of amyloid beta (Aβ) in senile plaques in the brain. Aβ accumulates in nerve cells, causing cellular dysfunction through various mechanisms, including mitochondrial dysfunction, leading to apoptosis. AD cell models are being studied to elucidate the neurotoxic effect of Aβ; however, conventional methods such as immunostaining and fluorescent molecular labeling require extensive sample manipulation and may produce artifacts not reflective of natural states. In this study, we employed Raman microscopy on AD cell models and simultaneously detected Aβ accumulated in cells and associated mitochondrial dysfunction through cytochrome distribution changes. This label-free method enables the observation of AD cell models in a more natural state and is expected to contribute to elucidating the mechanism of cell damage caused by Aβ. Furthermore, applying this system to experiments involving Aβ inhibitor administration enables rapid, label-free evaluation of drug activity, which is highly valuable in drug development.

## Graphical abstract

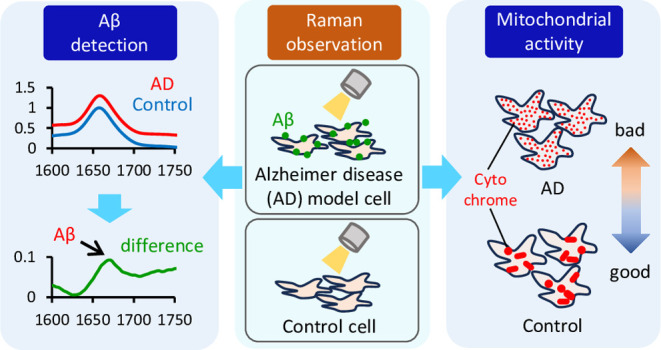

## Introduction

Alzheimer’s disease (AD) is the leading cause of dementia,^1^ with the formation of senile plaques composed of accumulated amyloid-β (Aβ) in the brain being its most characteristic feature.^2^ Aβ, especially Aβ oligomers and protofibrils, bind to neurons, causing cellular dysfunction and ultimately apoptosis through various mechanisms, including synaptic damage, mitochondrial damage, oxidative stress, and cell membrane damage.^3^ To elucidate the mechanism of these cellular dysfunctions, AD cell models are generated by exposing neurons to Aβ. Conventional methods use immunostaining after cell fixation,^4,5^ however, this alters sample properties due to processing and reagent binding. Alternatively, fluorescently labeled Aβ has been employed,^6,7^ yet labeling is thought to alter kinetics during Aβ aggregation and cell binding, raising concerns that the observed phenomena differ from those occurring with unlabeled Aβ. Therefore, techniques allowing observation of Aβ accumulation and cellular changes under near-physiological conditions are needed.

Raman microscopy detects Raman scattering generated at specific molecular vibration frequencies when biological samples are irradiated with excitation light. This enables noninvasive, label-free measurement of molecular distributions within samples.^8–10^ Previous studies have applied Raman microscopy to observe Aβ in senile plaques of post-mortem brain tissue,^11–16^ however, none have detected Aβ at the cellular level. In this study, we observed AD cell models with Raman microscopy to detect Aβ cell accumulation.

Detection of cellular dysfunction associated with Aβ accumulation is essential for elucidating the mechanism of Aβ neurotoxicity. In particular, mitochondrial dysfunction is considered an important hallmark in AD.^17,18^ Aβ accumulation causes mitochondrial dysfunction by generating oxidative stress, inhibiting electron transfer, and depleting ATP.^19–21^ Common assays for mitochondrial dysfunction—such as MTT, Alamar blue, ATP, and mitochondrial membrane potential detection (MitoTracker, JC-1)—are limited by cell damage due to measurement operations, lack of single-cell resolution, different physiological conditions for staining manipulations, and time-consuming measurements. Therefore, a more convenient method for measuring mitochondrial dysfunction at the single-cell level in physiological conditions is desired, and Raman microscopy is a promising candidate.

One approach focuses on the mitochondrial protein cytochrome, with our previous study proving that its Raman intensity reflects mitochondrial activity in a glutamate-induced neuronal cell death model.^22^ Furthermore, Raman microscopy can evaluate cellular activities such as intracellular mitochondrial function and oxidative stress status,^22^ and evaluate the progression of apoptosis in apoptotic cells by imaging the process of cytochrome efflux from mitochondria to the cytoplasm.^23^

In this study, we examined changes in cytochrome distribution to evaluate mitochondrial dysfunction in AD cell models. Our aim was to establish a method for simultaneously detecting Aβ accumulation in cells and associated mitochondrial dysfunction using Raman microscopy on AD cell models. This approach enables the observation of AD cell models in a more natural state without labeling, offering new insights into Aβ-induced cell damage. Furthermore, applying this system during Aβ inhibitor administration to examine the inhibitor-induced decrease in Aβ accumulation and suppression of cellular dysfunction, could allow label-free and rapid evaluation of drug activity, supporting drug development.

## Experimental Section

### Cell Culture

In this study, we used the human neuroblastoma SH-SY5Y cell line, the most commonly used model in AD research. SH-SY5Y cells were cultured in a 1:1 mixture of Eagle’s Minimum Essential Media (EMEM, FUJIFILM Wako Pure Chemical) and Ham’s F-12 (FUJIFILM Wako Pure Chemical), supplemented with 10% FBS, and antibiotic–antimycotic mixed stock solution (Nacalai Tesque). Cells were maintained at 37 °C in a humidified atmosphere containing 95% air and 5% CO_2_. Neuron differentiation followed the method of Encinas et al.^24^ Cells were plated at a density of 6.0 × 10^4^ cells/cm^2^ in culture dishes (Corning) or quartz dishes (FPI) coated with type I collagen (FUJIFILM Wako Pure Chemical). From day 1 after plating, cells were differentiated with 10 μM all trans-retinoic acid (FUJIFILM Wako Pure Chemical) for 5 days in the cell medium containing 15% FBS. Then, cells were switched to serum-free medium supplemented with 50 ng/mL BDNF (FUJIFILM Wako Pure Chemical) and were grown for 5 days. Neural differentiation was confirmed by observing the expression of neuron-specific markers (Figure S1). After differentiation, SH-SY5Y cells were coincubated with 10 μM Aβ oligomer for 24 h and fixed with 4% PFA for 15 min at room temperature.

### Aβ Oligomer Preparation

Aβ (human, 1–42) was purchased from PEPTIDE INSTITUTE, INC. Aβ oligomers were prepared according to Stine et al.^25^ Briefly, Aβ was added to hexafluoroisopropanol (HFIP) to obtain a homogeneous monomeric solution, which was allowed to stand for 30 min, aliquoted into Eppendorf tubes, and left on a clean bench overnight to evaporate HFIP. The solution was then freeze-dried in a lyophilizer (JFD320, Nihon-denshi) and stored at – 30 °C until use.

For use, the peptide was reconstituted to 1 mM in DMSO after bringing to room temperature, vortexed for 30 s, sonicated in a bath sonicator for 10 min, diluted in 20 mM HEPES to 100 μM, vortexed for 15 s, and incubated at 4 °C for 24 h. The Aβ oligomer solution was then dissolved in EMEM/Ham’s F-12 with 50 ng/mL BDNF and applied to cells at a final concentration of 10 μM.

### Slit-Scanning Raman Microscopy

Hyperspectral Raman scattering images were obtained using a home-built slit-scanning Raman microscope. Excitation at 532 nm was generated by a frequency-doubled Nd/YVO_4_ laser (Verdi, Coherent Inc.) with a power density of 5 mW/μm^2^ at the sample plane. The laser beam was shaped into a line by a cylindrical lens and focused on the cells at the microscope stage with a water immersion objective lens (CFI75 Apochromat 25×, NA 1.1; Nikon). Backscattered Raman signals were collected by the same objective lens and passed through a 532 nm long-pass edge filter (LP03-532RU-25, Semrock) before entering a spectrograph (MK-300, Bunkoh Keiki, Co, Ltd.) to block the excitation light and Rayleigh-scattered light. Raman signals were detected with a cooled Charge coupled device (CCD) camera (Pixis 400B, Princeton Instruments) at 5 s exposure per line. A single-axis galvanometer mirror was used to scan 63 lines per image, yielding 25200 spectra (400 pixels × 63 lines) per image, with an image resolution of 0.635 μm/pixel.

### Raman Data Analysis

Hyperspectral Raman images were acquired following previously described methods.^9,23,26^ After acquisition, cosmic rays were removed and singular value decomposition (SVD) was performed to extract the loading vectors contributing to image contrast. Raman images were generated from peak intensities at selected wavenumbers after background subtraction. Regions of interest (ROIs) for cells were defined manually based on composite images at 1685 cm^–1^ (protein) and 2852 cm^–1^ (lipid).

Spectra from 550 to 3026 cm^–1^ were corrected via polyfit fluorescence background removal after SVD. Cell-derived spectra were calculated by subtracting spectra outside the ROI (quartz and other background) from those inside the ROI.

For spectra in amide I region, moving-average processing was applied, followed by background subtraction via polyfit fluorescence removal (first order) over the 1520–1800 cm^–1^ range (Figure S2a). Images of 400 × 63 pixels were acquired at multiple locations within each sample. Cell-region ROIs were determined using the method described above, and cell-derived spectra for each image were generated by subtracting the mean spectra outside the cellular regions from the mean spectra within the cellular regions. The resulting cell-derived spectra were then normalized to the amide I peak (Figure S2b). Because the Aβ β-sheet peak (1670 cm^–1^) overlapped the amide I shoulder and was difficult to confirm in the original cell-derived spectrum, it was isolated by difference spectra between the Aβ-treated and control groups. Aβ Raman intensity was quantified as follows: The second derivative of the amide I spectrum was calculated to identify the inflection point, a straight line was drawn from the amide I peak to the inflection point on the right side, and the vertical distance at 1670 cm^–1^ was defined as the Aβ Raman intensity (Figure S2c).

### Immunostaining

Differentiated SH-SY5Y cells were treated with 10 μM Aβ oligomers and incubated for 24 h, then fixed in 4% PFA. The cells were then incubated in TBS containing 0.3% Triton X-100 and 5% donkey serum for 1 h at room temperature, followed by incubation with the primary antibody solution (in TBS containing 0.3% Triton X-100 and 1% donkey serum) overnight at 4 °C. After washing with PBS, the secondary antibody solution (in TBS containing 0.3% Triton X-100 and 1% donkey serum) was added and incubated overnight at 4 °C. After washing with PBS, the samples were incubated with Hoechst 33342 for 15 min at room temperature for nuclear staining. Immunostained samples were observed with a confocal laser scanning microscope (FLUOVIEW FV3000; Evident Corp., Tokyo, Japan) with an objective lens (LUCPlanFL N 20×, NA 0.7; Olympus). Images were acquired at a resolution of 0.311 μm/pixel. The primary and secondary antibodies are listed in Tables S1 and S2.

### Cytochrome Image Analysis

In this study, we focused on changes in cytochrome distribution as an indicator of cellular dysfunction. Cytochrome is a mitochondrial protein that is released from mitochondria into the cytoplasm during apoptosis.^27^ Thus, its distribution changes from a punctate distribution localized in mitochondria to a diffuse distribution throughout the cell. To quantify this change, the punctate/diffuse index has been introduced,^28^ calculated as the standard deviation of the average brightness of all the pixels in the intracellular region. As cytochrome diffuses into the cytoplasm during apoptosis, the punctate/diffuse index decreases since the brightness of the pixels in the cell is evenly distributed. This method was employed to both immunostained and Raman cytochrome images. The diffusion index was calculated by dividing the standard deviation of the intracellular region intensity by the mean intensity of the intracellular region, as described in eq 1.

$$diffusion\;index=\frac{s\tan dard\;deviation\;inside\;ROI}{mean\;intensity\;inside\;ROI}$$ 1

### MitoTracker Staining

Differentiated SH-SY5Y cells were treated with 500 nM MitoTracker Red CMXRos (Invitrogen) and incubated at 37 °C with 5% CO_2_ for 45 min. Cells were fixed in 4% PFA for 15 min at room temperature and observed using a confocal laser scanning microscope (FLUOVIEW FV3000) with a 20× objective lens (excitation 579 nm, emission 599 nm). MitoTracker luminance was calculated by manually creating ROIs based on tubulin immunostaining and calculating the average MitoTracker luminance within each ROI.

### Alamar Blue Assay

Mitochondrial function was assessed in cells using the Alamar Blue Cell Viability Reagent (Invitrogen). Differentiated SH-SY5Y cells seeded in 96-well plates were treated with 10 μL of Alamar blue reagent per 100 μL of medium and incubated at 37 °C for 24 h. Fluorescence was measured with a plate reader (SH-9000Lab, Hitachi) at 550 nm excitation and 600 nm emission.

### TUNEL Assay

Cell apoptosis was detected using the In Situ Cell Death Detection Kit, Fluorescein (Roche). Cells were fixed in 4% PFA for 15 min at room temperature and permeabilized in 0.1% TritonX-100 dissolved in 0.1% sodium citrate at 4 °C for 2 min. Then, they were incubated in TUNEL reaction mixture at 37 °C for 1 h, followed by Hoechst 33342 staining and observation with a confocal microscope. Hoechst was detected in the blue channel and TUNEL in the green channel. TUNEL-positive cell counts were performed manually, and Hoechst-positive cell counts were performed after segmentation using the default cyto3 model in Cellpose 3.0.6. Apoptosis was expressed as the ratio of the number of TUNEL-positive cells to the number of Hoechst-positive cells.

### RT-qPCR (Reverse Transcription Polymerase Chain Reaction)

RNA was extracted from cells using Sepasol-RNA I Super G (Nacalai Tesque). Complementary DNA (cDNA) was synthesized from 1 μg of RNA via reverse transcription using SuperScript IV VILO Master Mix (Invitrogen) according to the manufacturer’s instructions. RT-qPCR reactions were performed on a 7500 Fast Real-Time PCR system using THUNDERBIRD SYBR qPCR Mix (TOYOBO). The reaction conditions were 95 °C for 20 s, followed by 40 cycles at 95 °C for 3 s and 60 °C for 30 s. Relative gene expression levels were determined by the 2-ΔΔCt method relative to GAPDH. Primer sequences are shown in Table S3.

### Immunodepletion with Aβ Inhibitory Antibodies

Immunodepletion is a method for isolating target molecules from solution and is also used in Aβ cytotoxicity investigations.^29,30^ Lecanemab (Selleck) was used as the Aβ inhibitory antibody, and Human IgG4 isotype control (Selleck) as the control antibody without Aβ inhibition. Recombinant Protein G beads (25 μL; Dynabeads, Invitrogen) were transferred into an Eppendorf tube, collected using a magnet, and the supernatant was removed. Lecanemab or the Human IgG4 isotype control dissolved in 100 μL of PBS to a concentration of 0.5 μM was added to Dynabeads and gently agitated with a rotator for 30 min at room temperature for antibody binding. After collecting Dynabeads using a magnet and removing the supernatant, Dynabeads were washed with 100 μL of PBS, then incubated with 100 μL of 10 μM Aβ oligomers dissolved in EMEM/Ham’s F-12 medium supplemented with 50 ng/mL BDNF. Dynabeads were gently agitated with a rotator for 30 min at room temperature to remove the Aβ oligomers from the medium. Dynabeads were collected by a magnet and the supernatant was applied to differentiated SH-SY5Y cells.

### ELISA

ELISA was employed to quantify Aβ using the Human Amyloidβ­(1-42) (FL) Assay Kit (Immuno-Biological Laboratories) and confirm that Aβ was completely removed, following the manufacturer’s instructions. Briefly, 100 μL of the sample was placed in an antibody-coated plate and incubated overnight at 4 °C. After washing with the washing buffer, 100 μL of labeled antibody was added and incubated at 4 °C for 1 h. After washing with the washing buffer, 100 μL of the substrate solution was added and incubated at room temperature for 30 min. After adding 100 μL of stopping solution, the absorbance was measured at 450 nm using a plate reader (SH-9000Lab, Hitachi). The absorbance was measured for each of three Lecanemab- and IgG4-treated samples.

### Western Blot

The Aβ oligomer solution (100 μM) was diluted with H_2_O and then adjusted to a final concentration of 10 μM using the sample buffer solution without the reducing reagent. Each sample (10 μL) was loaded to SuperSep Ace 5–20% 17-well gels (Wako) and electrophoresed for 1 h at 200 V. The samples were then transferred to PVDF membranes (Transblot Turbo transfer pack PVDF mini, Bio-Rad, 1704156), washed twice with Tris-buffered saline/Tween-20 (TBS-T), and shaken in 5% skim milk dissolved in TBS-T for 60 min at room temperature. After washing three times with TBS-T, the membranes were incubated overnight at 4 °C with 11A1 antibody diluted 100-fold in 1% BSA TBS-T. After washing three times with TBS-T, the membranes were incubated with horseradish peroxidase-conjugated antimouse secondary antibody diluted 5000-fold in 1% BSA TBS-T for 2 h at room temperature. After removal of the secondary antibody solution, the membranes were washed three times with TBS-T. Bands were detected with the Molecular Imager ChemiDoc XRS + system (Bio-Rad Laboratories) using ECL Prime Western Blotting Detection Reagent (Cytiva).

### Electron Microscopy

The Aβ oligomer solution (100 μM, 5 μL) was applied to MAXTAFORM Grid I (Graticules Optics) treated with Formvar film, carbon deposition, and hydrophilization. After 2 min, the grid was replaced with 1% uranyl acetate and air-dried. Images were taken with a transmission electron microscope (HT7800, Hitachi).

Differentiated SH-SY5Y cells treated with 10 μM Aβ oligomer and cultured for 24 h were fixed in 2% glutaraldehyde/0.1 M phosphate buffer (pH 7.4), followed by 2% osmium tetroxide for 2 h at 4 °C. Dehydration was performed in a graded ethanol series (30–100%, 15 min each) at room temperature. Embedding was performed in Quetol 812 epoxy resin, polymerized at 60 °C for 48 h. Ultrathin slices (80–90 nm) were cut with an ultramicrotome. Carbon vacuum deposition was employed for coating, and double electron staining was performed with uranyl acetate/lead staining solution. Imaging was performed on a JEM-1400 (JEOL Co. Ltd.) electron microscope operated at 100 kV. Sample preparation and electron microscopy observation were carried out by the Hanaichi UltraStructure Research Institute (Aichi, Japan).

### Statistical Analysis

All data are expressed as the mean ± SEM. Comparisons between two groups were performed using Student’s *t*-test. *P* < 0.05 was considered statistically significant for all tests.

## Results and Discussion

### Confirmation of Aβ Oligomer Formation and Raman Spectra

The formation of Aβ oligomers was first confirmed by Western Blot; as shown in Figure 1a, multiple bands were observed in the 18–200 kDa region, indicating Aβ oligomers of multiple molecular weights. Transmission electron microscopy (TEM) further confirmed oligomer formation, showing several spherical Aβ structures (Figure 1b).

**Figure 1:**
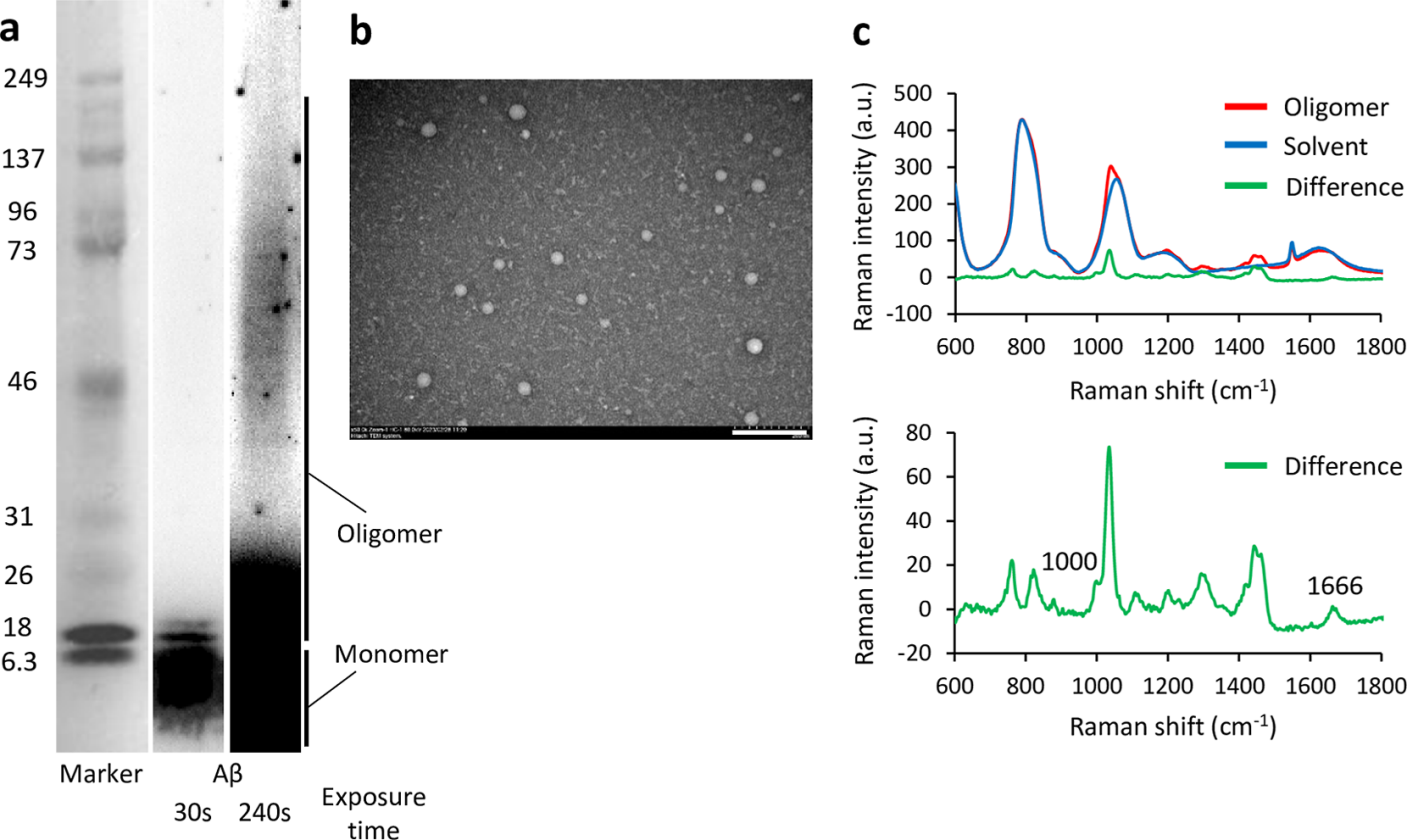
Confirmation of Aβ oligomer formation and Raman spectra. (a) Western blots (low and high exposure times) of Aβ oligomers showing multiple bands (8–200 kDa), consistent with Aβ oligomers of different molecular weights. (b) Electron micrograph of Aβ oligomers revealing several spherical Aβ oligomers. Scale bar: 200 nm. (c) Raman spectra of Aβ oligomers (10 μL) and solvent control, with the difference spectrum revealing a peak at 1666 cm^–1^, characteristic of the β-sheet structure of Aβ. Other peaks were attributed to HEPES and DMSO (Figure S3).

The Raman spectra of the Aβ oligomers, solvent (HEPES and DMSO), and the difference spectrum between the two are shown in Figure 1c. The majority of peaks in the difference spectrum originated from the solvent (Figure S3), while the peaks at 1666 and 1000 cm^–1^ were attributed to the Aβ oligomers. The peak at 1666 cm^–1^ corresponds to C=O stretching of the β-sheet structure in Aβ, a well-established Aβ indicator in several previous studies. Therefore, Aβ oligomers were successfully formed and characterized, and the 1666 cm^–1^ peak was used to detect Aβ in subsequent experiments.

### Aβ Accumulation Detection in AD Cell Models via Raman Microscopy

AD cell models (Aβ group) were generated by incubating differentiated SH-SY5Y cells with 10 μM Aβ oligomers (Figure 2a). SH-SY5Y cells were chosen as they are widely used in AD research,^4,21,31,32^ and Aβ oligomers were used because of their high neurotoxicity, known to induce mitochondrial dysfunction, oxidative stress, excessive Ca influx, and cell membrane damage, being important in AD pathogenesis.^33^ Comparisons were performed against the control group, treated with solvent only.

**Figure 2:**
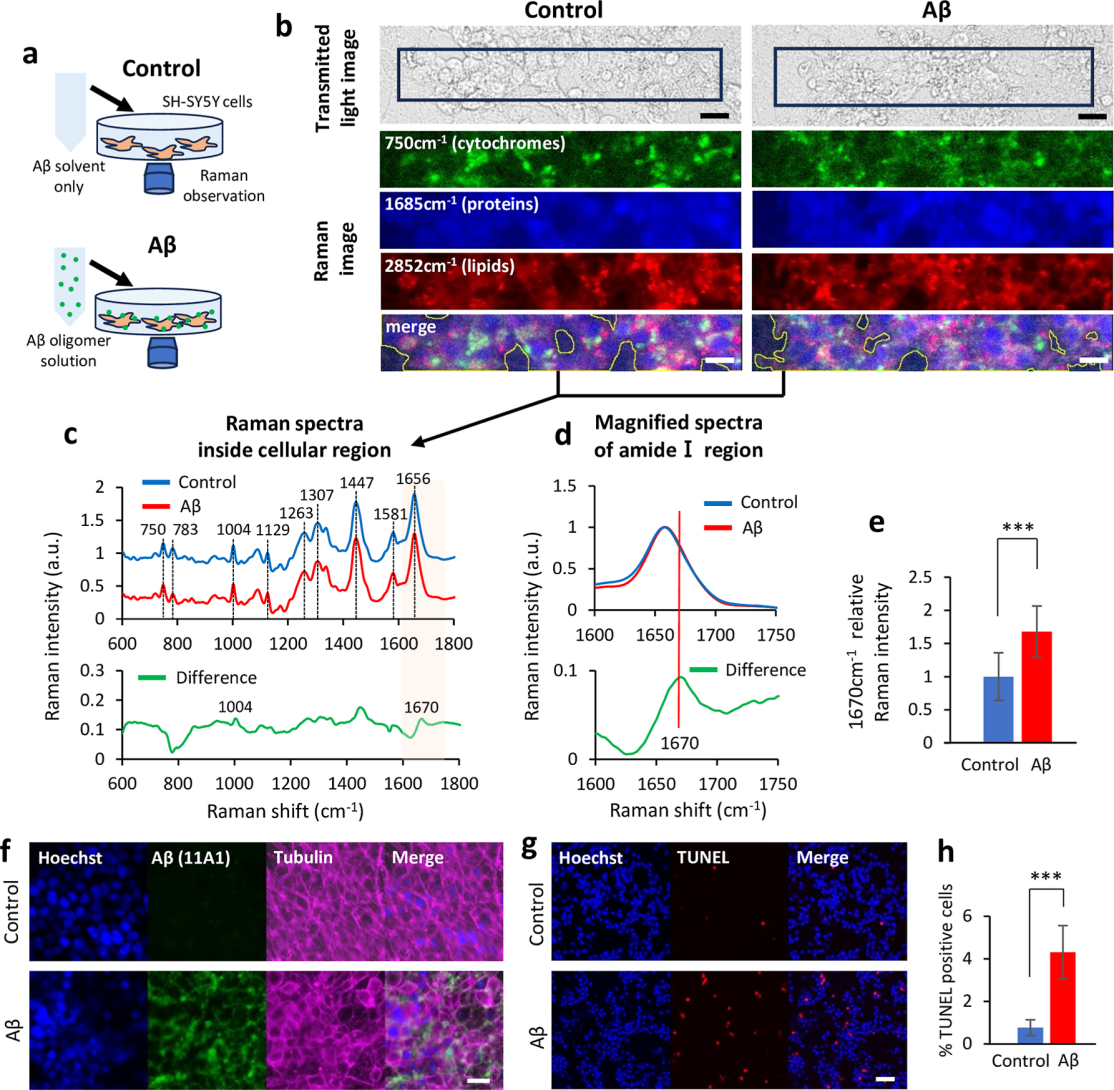
Raman microscopy revealing Aβ accumulation in AD model cells. (a) Schematic of the AD cell model; differentiated SH-SY5Y cells were treated with 10 μM Aβ oligomer for 24 h, whereas control cells received solvent only. (b) Raman images of the AD cell model revealing cytochromes (750 cm^–1^), proteins (1685 cm^–1^), and lipids (2852 cm^–1^), with a merge image. Raman microscopy was performed on the region outlined in the transmitted light image. Scale bar: 20 μm. (c) Raman spectra of the AD cell model measured within the cellular region (yellow outline in b), with the difference spectrum (Aβ–control group spectra) revealing peaks at 1670 cm^–1^ (β-sheet of Aβ) and 1004 cm^–1^ (phenylalanine). Spectra represent averages from intracellular ROIs across eight regions per sample. (d) Enlarged amide I region (shaded portion in c); although the 1670 cm^–1^ peak is obscured in the raw spectra, it is clearly visible in the difference spectrum. (e) Relative Raman intensities at 1670 cm^–1^ with the Aβ group showing a significantly higher intensity. Control: *N* = 29 images (Sample 1:8, Sample 2:8, Sample 3:7, Sample 4:6); Aβ: *N* = 29 images (Sample 1:8, Sample 2:8, Sample 3:6, Sample 4:7). ***P < 0.001, Student’s *t*-test. (f) Immunostaining with Aβ oligomeric antibody 11A1, confirming Aβ accumulation in cells and nerve fibers of Aβ-treated cells. Scale bar: 20 μm. (g,h) Confirmation of apoptosis in the Aβ group via TUNEL staining. The proportion of TUNEL-positive cells among Hoechst-positive cells was significantly higher in the Aβ group compared to the control group 1 day after Aβ administration. Scale bar: 50 μm. *N* = 20 images per group (4 samples, 5 images each).

Transmitted light images (Figure S4) of the control group exhibited no change in cell morphology. However, the Aβ-treated group displayed irregular cell morphology and nerve fiber irregularities 1 day after treatment, followed by apoptosis and numerous remnants of shrunken cell bodies after 2 days. TUNEL staining image 24 h post-treatment revealed numerous TUNEL-positive cells (red), reflecting apoptosis in the Aβ group compared to the control group (Figure 2g). Quantification revealed a significantly higher percentage of TUNEL-positive cells (relative to Hoechst-stained cells) in the Aβ group compared with control (Figure 2h). For Raman observations, cells were examined 24 h after Aβ administration—when early morphological and apoptotic changes appear but before apoptosis is complete—consistent with many previous studies.

AD and control cells seeded on quartz Petri dishes were observed via slit-scanning confocal Raman microscopy at 532 nm. Raman images (400 × 63 pixels) were acquired from a 40 μm region (Figure 2b), with characteristic peaks including 750 cm^–1^ (pyrrole ring of cytochrome), 1685 cm^–1^ (protein amide I), and 2852 cm^–1^ (lipid CH_2_). ROIs of cellular regions were defined based on composite Raman images of proteins at 1685 cm^–1^ and lipids at 2852 cm^–1^, and Raman spectra within the cellular regions were averaged across eight sampling locations within the same sample (Figure 2c). Difference spectra (Aβ spectrum minus control spectrum) revealed a peak at 1670 cm^–1^ derived from the β-sheet, which is considered to be the most representative peak of Aβ, thereby confirming Aβ accumulation in treated cells. Notably, the lipid CH_2_ stretching band at 2852 cm^–1^ showed no detectable difference in intensity or spatial distribution between the Aβ and control groups (Figure S5), indicating that the increase at 1670 cm^–1^ does not arise from lipid-related spectral changes. The presence of a phenylalanine peak at 1004 cm^–1^—recognized as a protein-associated band—was also observed in the Aβ protein Raman spectrum, possibly deriving from Aβ.

As shown in Figure 2d, the peak at 1670 cm^–1^ is masked by the amide I band in the Aβ spectrum but becomes evident in the difference spectrum. The Raman intensity at 1670 cm^–1^ was quantified using the method shown in Figure S2c and found to be significantly higher in Aβ-treated cells than the control (Figure 2e), providing quantitative evidence of Aβ accumulation. Immunostaining with the Aβ oligomer antibody 11A1 (Figure 2f) confirmed these results: control cells showed no signal, whereas Aβ-treated cells exhibited 11A1 staining primarily in cell membranes and nerve fibers.

In this study, we demonstrate cellular-level detection of Aβ accumulation using Raman spectroscopy. Previous Raman investigations focused primarily on extracellular senile plaques in human^11–13^ and mouse^14–16^ brains tissue, whereas Aβ accumulation in neurons—considered central to AD pathogenesis and an early event in disease progression—had not been detected at the cellular level.^34,35^ Therefore, our successful detection of Aβ accumulation at the cellular level is of high significance not only for early AD detection but also for understanding AD pathogenesis.

Label-free Aβ detection via Raman microscopy is another key achievement of our study. Several previous studies on the kinetics of Aβ in AD cell models have relied on fluorescently labeled Aβ (using TMR, Alexa Fluor, quantum dots),^6,7,32^ or postfixation immunostaining,^4^ which may alter aggregation behavior or disturb physiological conditions. Raman microscopy, on the other hand, enables direct, label-free observation, making it well-suited for detecting Aβ dynamics in living cells under near-physiological conditions.

In this study, we focused on the 1670 cm^–1^ peak derived from β-sheet structures in proteins, being widely used as an Aβ marker.^12–16^ The amide I band (1650 cm^–1^), attributed to the stretching vibration of the C=O peptidic bond, is sensitive to changes in protein secondary structures, with α-helix and β-sheet components appearing near 1658 and 1670 cm^–1^, respectively. Because AD pathology is characterized by aggregated β-sheet-rich proteins, the amide I peak shifts to 1670 cm^–1^ in affected regions. The Raman spectrum of the administered Aβ oligomers showed a peak at 1666 cm^–1^, consistent with previous studies. A phenylalanine peak at 1000 cm^–1^ was also observed, however, this feature is common to many proteins and is therefore not specific for Aβ. In the raw spectra of the AD model cells, no peak shift appeared at 1670 cm^–1^; however, it was observed in the difference spectra. This is possibly due to the less dense Aβ in the AD cells compared with the highly aggregated and dense Aβ in senile plaques; the peak at 1670 cm^–1^ is buried in the amide I spectra and only appears when the difference from the control group is taken. The polymerization state of Aβ may also be involved. Supporting this, Aβ fibrils produced stronger Raman intensity at 1670 cm^–1^ than oligomers (Figure S6), consistent with their high β-sheet content. Since most Aβ in senile plaques is fibril, whereas in AD model cells is thought to be oligomeric, the Raman intensity at 1670 cm^–1^ in AD model cells is probably lower. Aβ oligomers are considered the most neurotoxic Aβ species and are deeply related to AD pathogenesis. Therefore, detecting them in our model is of particular significance.

Spatial imaging of Aβ distribution was not achieved, likely due to the small amount and low degree of polymerization of Aβ accumulated in AD model cells, producing a weak 1670 cm^–1^ signal that was obscured by the strong amide I background. Although spontaneous Raman microscopy enables label-free observation, its sensitivity to weak signals is inherently limited. More advanced methods such as coherent anti-Stokes Raman scattering (CARS) may enable visualization of Aβ spatial distribution in future studies.^36^

### Mitochondrial Dysfunction Detection via Raman Microscopy

The potential of Raman spectroscopy to detect mitochondrial dysfunction in AD cell models was next examined. Previous studies have shown that cytochrome Raman intensity and distribution reflect mitochondrial function and apoptosis progression.^22,23^ Therefore, we focused on cytochrome as an indicator.

The Raman intensity of cytochrome at 750 cm^–1^ was compared between the Aβ and control groups, but no significant difference was observed (Figure S7). However, Raman imaging revealed clear distribution changes (Figure 3a), consistent with immunofluorescence images of the same region (Figure 3c). In controls, cytochrome showed a localization corresponding to mitochondria, whereas Aβ-treated cells showed diffuse distribution. Plot profiles of Raman and immunofluorescence images (Figure 3b, d) confirmed this pattern: in the control group, the spike-like increase in intensity reflects the punctate distribution of cytochrome, whereas the flattened profiles in the Aβ group indicate cytochrome diffusion. Owing to the optical and sampling characteristics of the Raman measurements, together with the broader molecular sensitivity of Raman scattering and potential perturbations introduced by immunostaining, cytochrome-positive regions appear slightly larger in Raman images than in corresponding immunofluorescence images (Supplementary Note 1). These minor differences did not significantly affect the analysis of cytochrome distribution, and the resolution of the Raman images was sufficient for this purpose.

**Figure 3:**
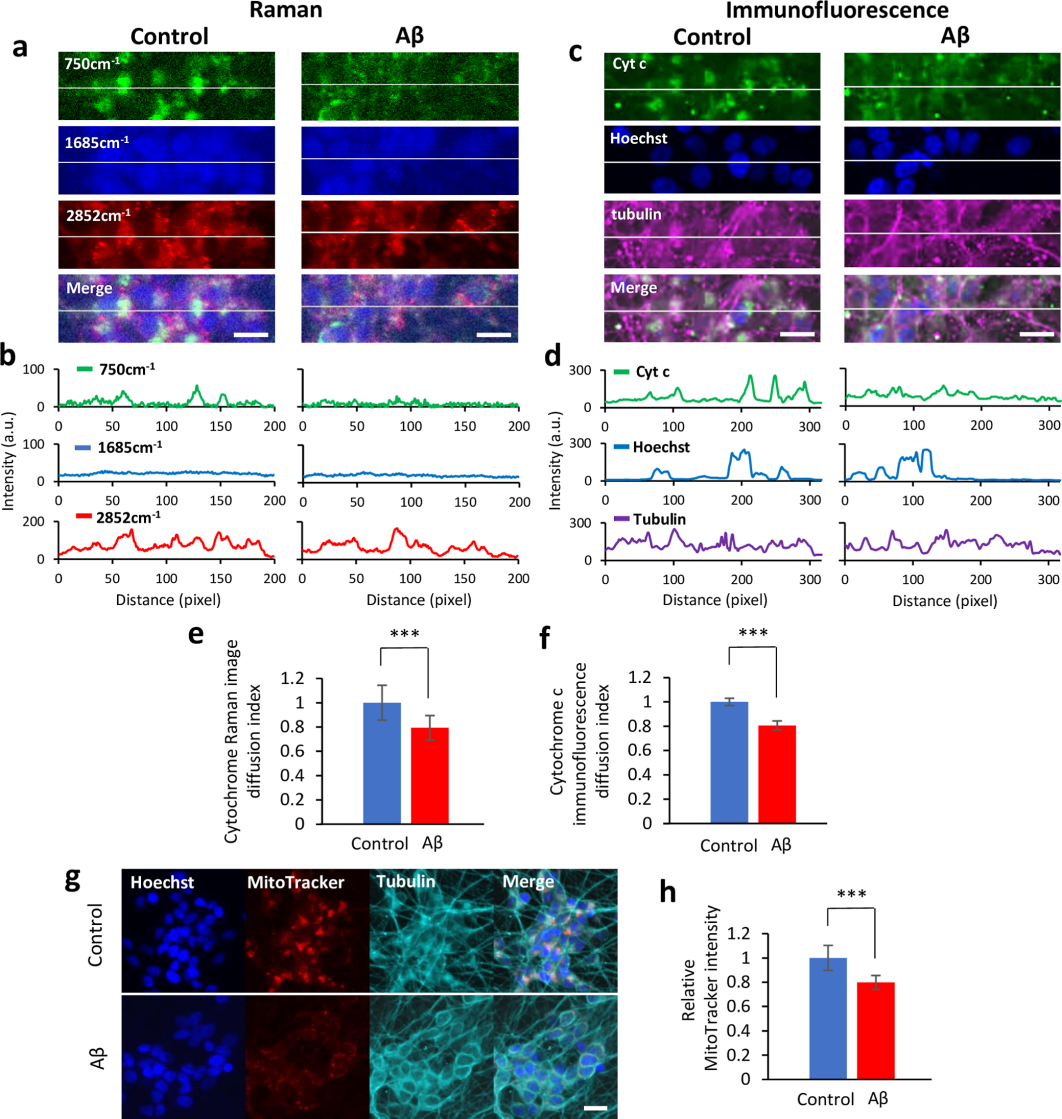
Detection of mitochondrial dysfunction in AD model cells via Raman microscopy. (a) Raman and (c) immunofluorescence images of cytochrome. At 750 cm^–1^, cytochrome displays punctate localization consistent with mitochondria in the control group, whereas in Aβ-treated cells it is diffusely distributed throughout cells. Scale bar: 20 μm. (b, d) Plot profiles of (a) and (c), respectively. At 750 cm^–1^, controls show a spike-like increase in intensity, reflecting punctate cytochrome localization, whereas Aβ-treated cells show flat profiles reflecting the diffusion of cytochrome. (e,f) Diffusion index analysis of Raman and immunofluorescence images, respectively. Both indices are significantly lower in the Aβ group, reflecting cytochrome diffusion. Raman: Control: *N* = 29 images (Sample 1:8, Sample 2:8, Sample 3:7, Sample 4:6); Aβ: *N* = 29 images (Sample 1:8, Sample 2:8, Sample 3:6, Sample 4:7); ***P < 0.001, *t*-test. Immunofluorescence: *N* = 30 images (3 samples, 10 images each); ***P < 0.001, *t*-test. (g) MitoTracker image showing reduced MitoTracker staining in the Aβ group, reflecting reduced mitochondrial function. Scale bar: 20 μm. (h) Quantification of MitoTracker intensity in intracellular regions confirming significantly reduced intensity in the Aβ group; *N* = 30 images (3 samples, 10 images each); ***P < 0.001, *t*-test.

To quantify cytochrome diffusion, the diffusion index was calculated for each Raman and immunofluorescence image, and comparisons were made between the two groups. The diffusion index reflects the diffusion degree of cytochrome, with smaller values indicating cytochrome diffusion. Both analyses showed a significantly lower diffusion index in the Aβ group than in the control group (Figure 3e,f), quantitatively demonstrating that cytochrome diffuses in the Aβ group.

To investigate the cause of cytochrome diffusion, the mitochondrial activity and structure were assessed. MitoTracker staining revealed significantly reduced intensity in the Aβ group compared to the control group (Figure 3g,h). Alamar blue assays showed no significant difference 1 day after Aβ oligomer administration, however, the mitochondrial activity was significantly decreased in the Aβ group after 2 days (Figure S8). Therefore, mitochondrial dysfunction was confirmed.

The relationship between the spatial distribution of mitochondria and cytochrome was examined via costaining with cytochrome *c* and the mitochondrial marker COX4 (Figure S9). In the control group, COX4 and cytochrome *c* were colocalized in a punctate pattern, whereas in the Aβ group, mitochondria indicated by COX4 were fragmented and tended to diffuse throughout the cell with a reduced degree of colocalization between COX4 and cytochrome *c*, suggesting that cytochrome *c* was at least partially leaking from mitochondria.

Electron microscopy confirmed these findings (Figure 4). In controls, mitochondria were clustered with high density and clear cristae structures, whereas Aβ-treated cells exhibited fragmented and dispersed mitochondria with swelling, reduced density, and unclear cristae structures. Therefore, nanoscale observations confirmed destroyed mitochondrial structures along with mitochondrial dysfunction.

**Figure 4:**
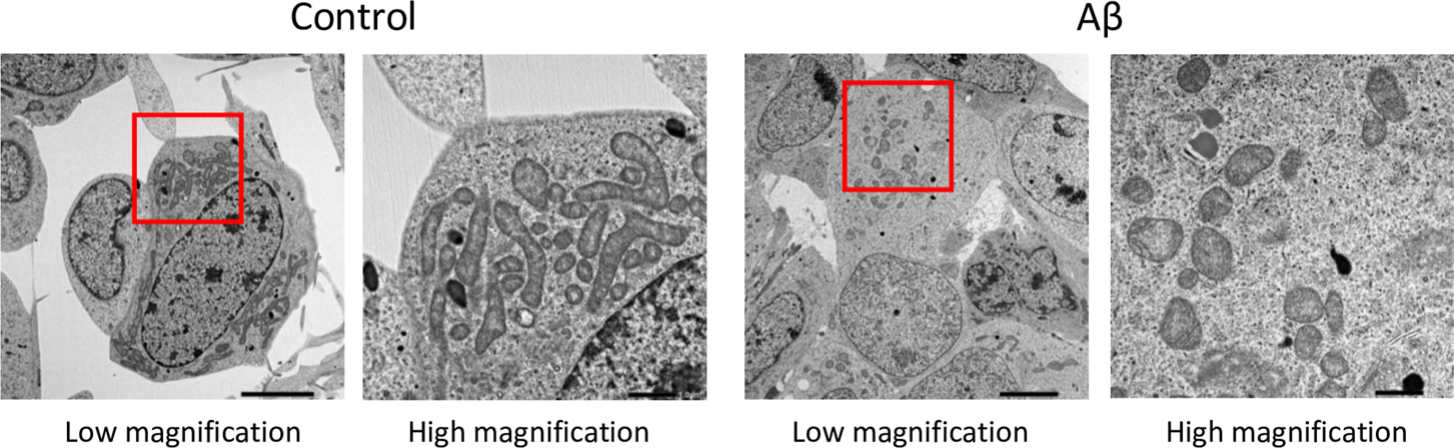
Transmission electron microscopy images revealing changes in mitochondrial morphology and distribution in AD model cells. The low magnification images (×1500) show clustered mitochondria for the control group and dispersed mitochondria within the cytoplasm for the Aβ group. The high magnification images (×5000) reveal denser mitochondria with visible cristae in the control group, and swollen mitochondria with lower density and destroyed cristae in the Aβ group. Scale bars: Low magnification images, 5 μm; high magnification images, 1 μm.

Together, these findings suggest that the distribution changes observed in Raman images—such as cytochrome diffusion throughout cells—result from mitochondrial fragmentation, network destruction, and cytochrome leakage into the cytoplasm during apoptosis. Thus, mitochondrial dysfunction caused by Aβ can be detected by Raman microscopy through changes in cytochrome spatial distribution.

Building on these observations, we interpret the changes in cytochrome distribution at 750 cm^–1^ in relation to mitochondrial function and apoptotic progression in AD model cells. Cytochrome, a key component of the mitochondrial electron transfer system, reflects mitochondrial activity and plays a central role in apoptosis through its release into the cytoplasm and subsequent caspase activation.^22,27^ Our previous Raman studies in apoptotic HeLa cells induced by Actinomycin D stimulation^23,26^ showed that cytochrome diffusion could be visualized via Raman microscopy as efflux from the mitochondria into the cytoplasm progressed. Under mild apoptotic conditions, the Raman intensity of cytochrome remained stable during the observation time scale (30 min),^23^ whereas under stronger apoptotic conditions, intensity decreased.^26^ This likely reflects the redox shift from reduced to oxidized cytochrome as apoptosis progresses. However, in early apoptosis, immediately after cytochrome is leaked from the mitochondria, no such change occurs. Consistent with this, we also observed a change in the distribution of cytochrome as it diffused throughout the cell, but no change in Raman intensity (Figure S7), indicating that reduced cytochrome diffused throughout the cells.

Two mechanisms may explain why cytochrome diffused throughout the cell in the AD model cells in this study. First, Aβ-induced cytotoxicity disrupted the mitochondrial network, causing mitochondrial fragmentation and intramitochondrial cytochrome diffusion throughout the cell. This was supported by COX4 immunostaining and electron microscopy, which revealed transformation from elongated tubular networks to fragmented, spherical mitochondria. In this case, cytochrome remains within the mitochondria with no change in redox state, explaining the stable Raman intensity. Second, cytochrome leakage from structurally damaged mitochondria disrupted via Aβ may have contributed, as suggested by swollen mitochondria with obscured cristae observed by electron microscopy, consistent with previous AD studies.^21,37,38^ While some cytochrome efflux may occur during apoptosis, the absence of Raman intensity changes suggests this was not a major factor in our model.

The Raman-based approach offers clear advantages over conventional assays for detecting mitochondrial dysfunction. Changes were detectable within 24 h of Aβ administration, earlier than Alamar blue (2 days) and comparable to MitoTracker staining (24 h), while providing label-free, single-cell resolution under physiological conditions without pretreatment.

In summary, the present study shows that Raman microscopy of AD model cells enables simultaneous label-free observation of Aβ accumulation and associated mitochondrial dysfunction, offering greater sensitivity and practicality over conventional methods.

### Detection of Lecanemab-Induced Inhibition of Aβ Accumulation via Raman Microscopy

Lecanemab is a humanized antibody with high selectivity for soluble Aβ aggregates (oligomers and protofibrils).^39,40^ Its administration to AD cell models is expected to inhibit Aβ cell binding and suppress Aβ-induced cell dysfunction. We therefore tested whether Raman microscopy could detect Lecanemab-mediated reduction in Aβ accumulation and suppression of mitochondrial dysfunction.

Aβ solutions immunodepleted with either Lecanemab or control IgG were administered to neurodifferentiated SH-SY5Y cells and cultured for 24 h. Aβ accumulation and mitochondrial dysfunction were compared between the two groups (Figure 5a). Prior to administration, Aβ concentration was measured using the ELISA method. The Aβ concentration in the Lecanemab group was significantly lower than that in the control IgG group, confirming the blocking effect of Lecanemab (Figure S10). Morphological analysis using transmitted light imaging revealed irregular cell and nerve fiber structures in the control IgG group, however, no such changes were observed in the Lecanemab group (Figure 5a). Consistently, TUNEL staining revealed significantly fewer apoptotic cells in the Lecanemab group (Figure 5e,f). Therefore, Lecanemab suppresses cell damage and apoptosis caused by Aβ.

**Figure 5:**
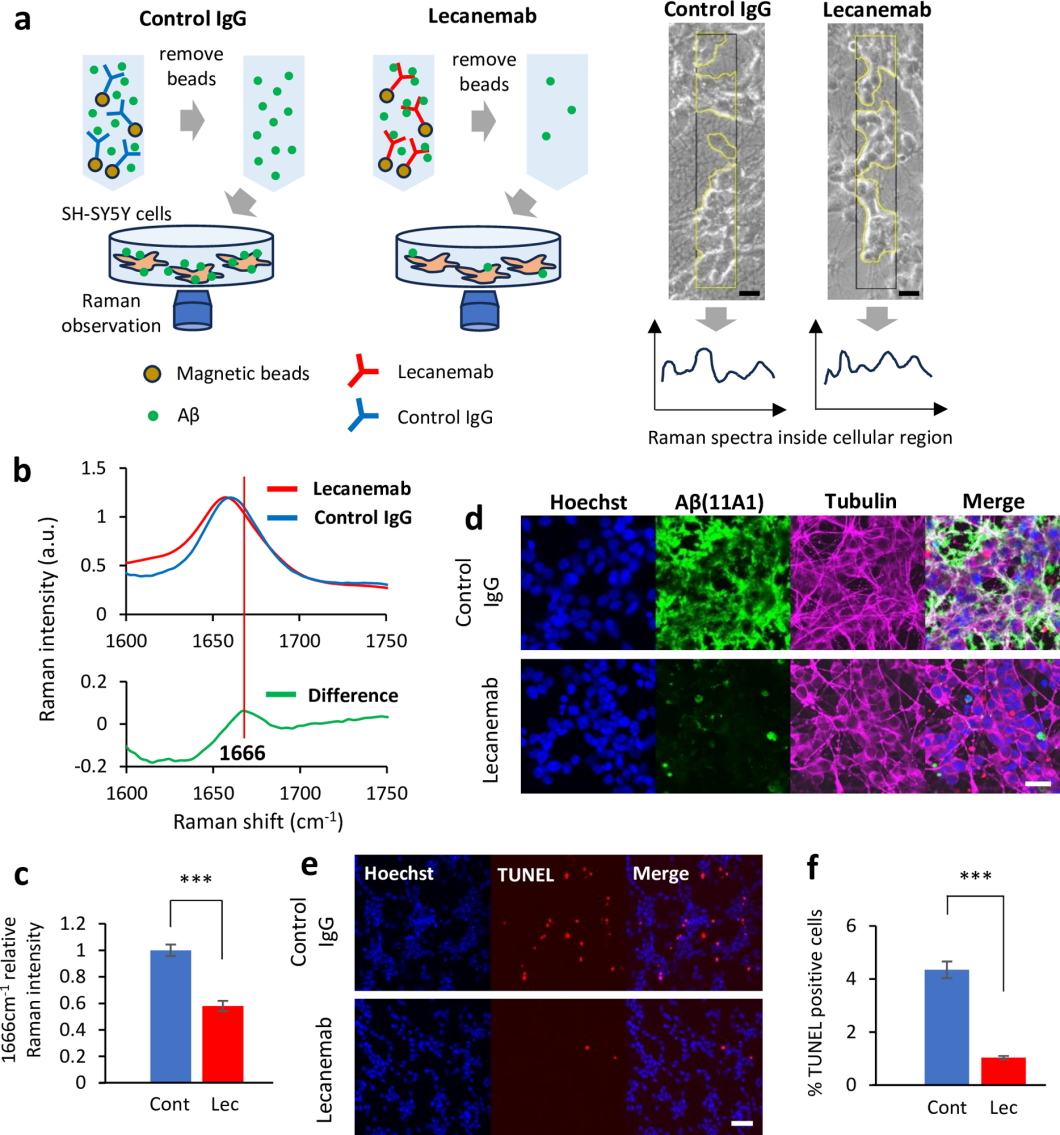
Detection of Lecanemab-induced reduction of Aβ accumulation in an AD cell model via Raman microscopy. (a) Schematic of immunodepletion where Lecanemab or control IgG-conjugated magnetic beads are mixed with the Aβ oligomer solution. Following magnetic removal of the beads, the supernatant is applied to SH-SY5Y cells. Lecanemab selectively binds Aβ in solution, whereas control IgG does not, resulting in reduced cellular Aβ accumulation only in samples immunodepleted with Lecanemab. Transmitted light images (upper right) showing irregular morphology of cells and nerve fibers in the control IgG group, but no specific changes in the Lecanemab group. Raman microscopy was performed on the 40 μm wide area (black frame), with spectra measured from the cellular region (yellow outline). Scale bar: 20 μm. (b) Raman spectra in the amide I region of cells exposed to Aβ pretreated with either Lecanemab or control IgG. Images (400 × 63 pixels) were acquired at six locations within each sample. Spectra from cellular regions were averaged, and representative mean spectra are shown. The lower panel shows the difference spectrum of the control IgG group minus the Lecanemab group, revealing the 1666 cm^–1^ peak and reflecting decreased Aβ accumulation in the Lecanemab group. (c) Quantification of 1666 cm^–1^ Raman intensity, revealing significantly lower intensity in the Lecanemab group compared with control. *N* = 18 images (3 samples, 6 images each). ***P < 0.001, *t*-test. (d) Immunostaining with Aβ oligomer antibody 11A1 showing marked accumulation in the control group and almost no accumulation in the Lecanemab group. Scale bar: 20 μm. (e, f) TUNEL assay results revealing a significantly lower proportion of TUNEL-positive cells relative to Hoechst-positive cells in the Lecanemab group. Scale bar: 50 μm. *N* = 15 images per group (3 samples, 5 images each). ***P < 0.001, *t*-test.

The difference spectrum (control IgG–Lecanemab group spectra) in the amide I region showed a β-sheet peak (1666 cm^–1^), reflecting reduced Aβ accumulation in Lecanemab-treated cells (Figure 5b). This peak was significantly reduced in the Lecanemab group compared to the + IgG group (Figure 5c), as measured using the method shown in Figure S2c.

To confirm these results, immunostaining was performed using the Aβ oligomer antibody 11A1 (Figure 5d). In the control IgG group, cells were abundantly stained with 11A1, whereas staining was minimal in the Lecanemab group. Therefore, it was concluded that the reduction in Aβ accumulation in AD cell models by Lecanemab could be detected using Raman microscopy.

### Detection of Lecanemab-Induced Suppression of Mitochondrial Dysfunction via Raman Microscopy

The potential of Raman microscopy to detect the mitigating effect of Lecanemab on mitochondrial dysfunction was next investigated via changes in cytochrome distribution at 750 cm^–1^ as an indicator of cellular dysfunction (Figure 6a). Immunofluorescence images of the same region are shown in Figure 6c. In the control IgG group, cytochrome showed diffuse cytoplasmic distribution throughout the cells, whereas in the Lecanemab group, it retained a punctate pattern reflecting the distribution of mitochondria.

**Figure 6:**
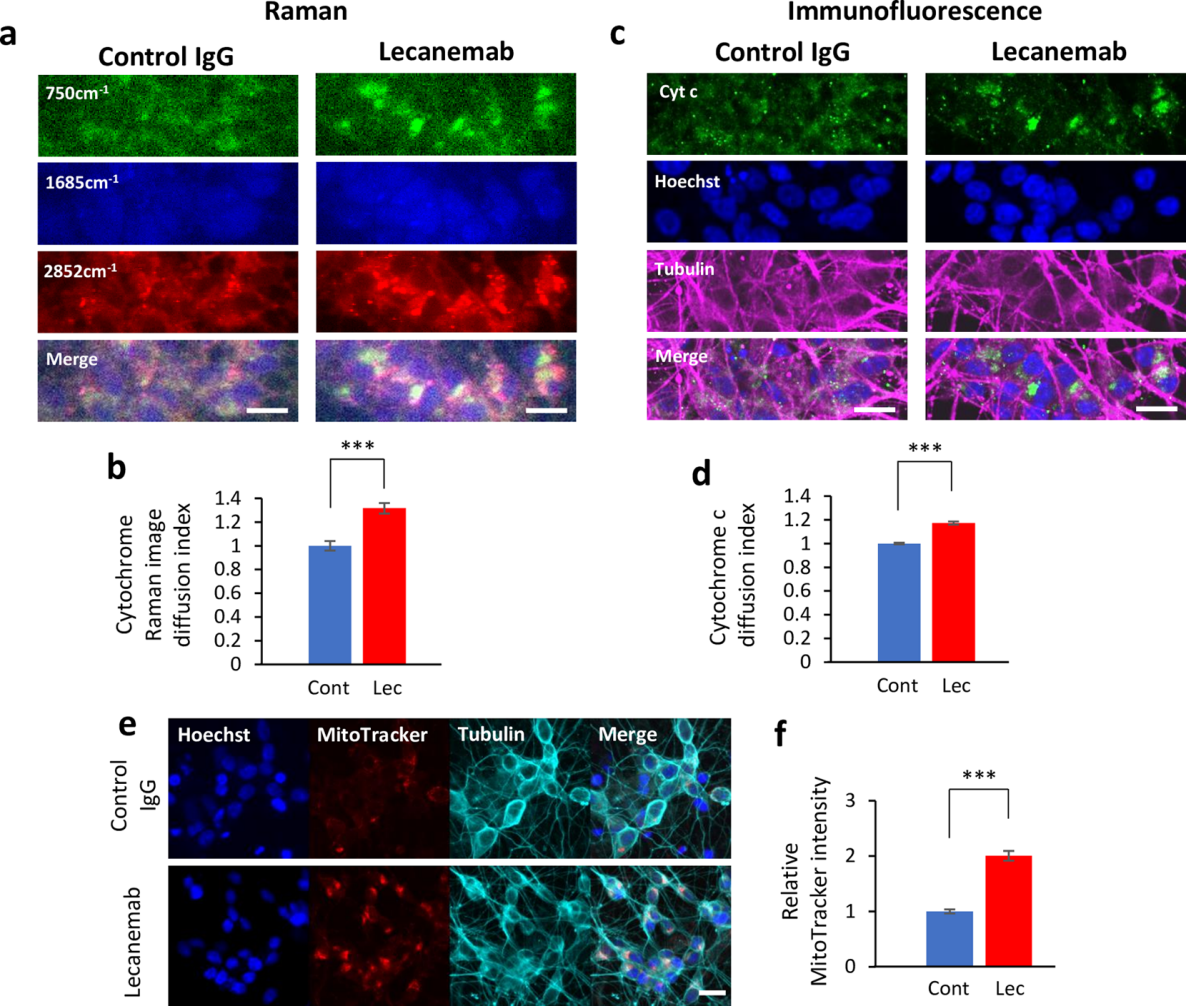
Detection of Lecanemab-induced cellular dysfunction inhibition via Raman microscopy. (a) Raman (750 cm^–1^) and (c) cytochrome c-immunostained images showing diffused cytochrome in the control IgG group and punctate distribution in the Lecanemab group. Scale bar: 20 μm. (b, d) Comparison of diffusion indices in cytochrome Raman and cytochrome *c* immunostaining images, respectively. Both indices are significantly higher in the Lecanemab group, reflecting the cytochrome diffusion inhibitory effect of Lecanemab. Raman: *N* = 18 images (3 samples, 6 images each); ***P < 0.001, *t*-test. Immunostaining: *N* = 24 images (3 samples, 8 images each); ***P < 0.001, *t*-test. (e) MitoTracker staining showing preserved mitochondrial intensity in the Lecanemab group compared with the control. Scale bar: 20 μm. (f) Quantification of MitoTracker intensity in the intracellular region revealing significantly higher intensity in the Lecanemab group. *N* = 15 images (3 samples, 5 images each); ***P < 0.001, *t*-test.

Quantification using the diffusion index confirmed this difference: values were significantly higher in the Lecanemab group (Figure 6b, d), confirming that the inhibitory effect of Lecanemab on the diffusion of cytochrome throughout the cell can be successfully detected via Raman microscopy.

To explore the mechanism of Lecanemab action, the mitochondrial function and structure were assessed. MitoTracker staining revealed significantly higher fluorescence in the Lecanemab group than in the control IgG group, indicating that mitochondrial dysfunction was suppressed in the Lecanemab group (Figure 6e,f). Co-staining of cytochrome *c* and mitochondrial marker COX4 (Figure S11) revealed diffuse COX4 distribution throughout the cells in the control IgG group, whereas cytochrome *c* was colocalized with COX4 in a punctate pattern in the Lecanemab group. Therefore, mitochondrial fragmentation was suppressed and cytochrome remained inside the mitochondria in the Lecanemab group.

Transmission electron microscopy corroborated these findings: Control cells exhibited swollen, dispersed mitochondria with reduced density and disrupted cristae, scattered throughout the cells. Conversely, Lecanemab-treated cells maintained dense, clustered mitochondria with clear cristae (Figure 7).

**Figure 7:**
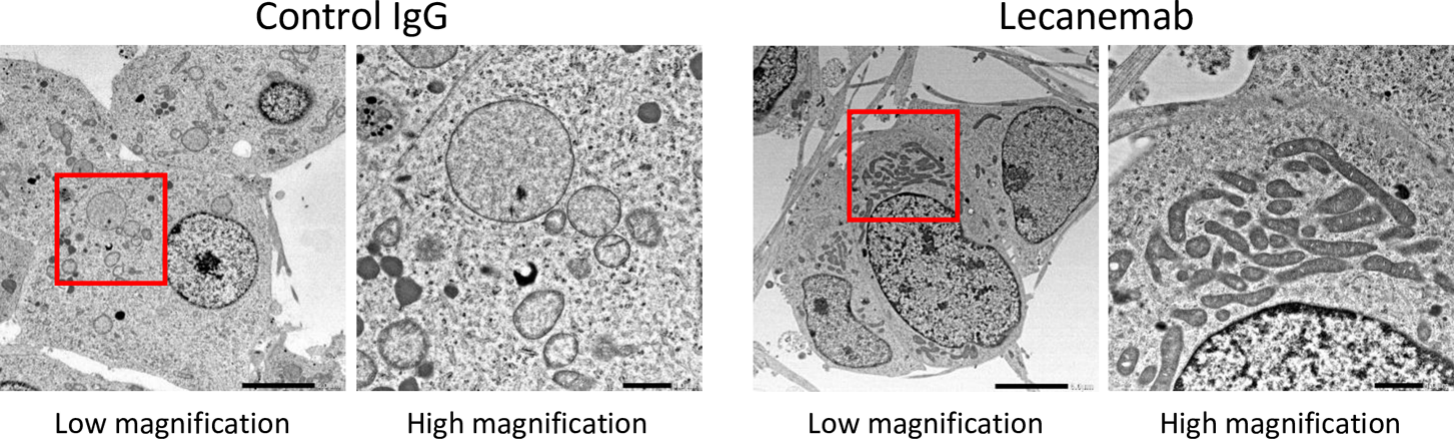
Transmission electron microscopy images, showing that Lecanemab preserves mitochondrial morphology and distribution. In the low magnification images (×1500), mitochondria are clustered in the Lecanemab group, whereas in the control IgG group, mitochondria are dispersed in the cytoplasm. In the high magnification images (×5000), mitochondria in the control IgG group are swollen, with low density, and destroyed cristae structures, whereas in the Lecanemab group, mitochondrial structures are maintained. Scale bars: Low magnification images, 5 μm; high magnification images, 1 μm.

Overall, these results indicate that Lecanemab suppressed mitochondrial fragmentation, diffusion, and cytochrome leakage, thereby preserving punctate cytochrome distribution. Raman microscopy can therefore detect the protective effect of Lecanemab on mitochondrial integrity and function in AD cell models.

### Implications of Lecanemab-Induced Reduction of Aβ Accumulation and Mitochondrial Dysfunction

Lecanemab, a humanized antibody derived from mouse mAb158 and highly selective for soluble Aβ aggregates, including oligomers and protofibrils,^39,40^ has been shown clinically to reduce amyloid burden and improve cognitive outcomes.^41^ In our AD cell model, Raman spectroscopy detected reduced cellular Aβ accumulation following Lecanemab treatment and preservation of punctate cytochrome distribution, indicating suppression of Aβ-induced mitochondrial dysfunction. Such findings suggest that Lecanemab prevents both mitochondrial fragmentation and cytochrome leakage associated with Aβ toxicity.

In AD drug development, drug efficacy is evaluated using AD cell models^42,43^ by measuring Aβ accumulation, mitochondrial dysfunction, and cell death, using staining-based or enzymatic assays, which are time-consuming, require labeling or fixation, and often lack single-cell resolution. Conversely, our method can detect mitochondrial dysfunction before cell death occurs, enabling label-free evaluation under near-physiological conditions in a relatively short time (2–3 h), and providing single-cell resolution. Methods for evaluating Aβ accumulation such as immunostaining or fluorescently labeled Aβ,^4,21,31^ also require staining or fixation, introducing nonphysiological conditions and adding time. Raman microscopy overcomes these limitations by allowing observation in a label-free state and having a short detection time.

Based on the above, this methodology offers a streamlined approach for evaluating drug efficacy in AD models without complex sample manipulation. While this study employed immunodepletion prior to cell exposure, future studies could incorporate Raman-tagged drugs to simultaneously examine drug dynamics in addition to changes in Aβ accumulation and mitochondrial dysfunction. This approach may enable more detailed analyses of the relationships between drug accumulation and Aβ accumulation, as well as drug accumulation and mitochondrial dysfunction.

## Conclusions

In this study, we employed Raman microscopy to observe AD cell models and successfully detected both cellular Aβ accumulation and associated mitochondrial dysfunction in a label-free manner. This method enables the observation of AD cell models in a more natural state without labeling and is expected to help elucidate mechanisms of Aβ-induced cell damage. Furthermore, its ability to rapidly evaluate drug activity without labeling highlights its potential as a valuable tool in drug development.

## Supplementary Material





## Data Availability

The data underlying this study are available in the published article and its Supporting Information.

## Notes

Confirmation of neural differentiation of SH-SY5Y cells; analysis methods of Raman spectra; comparison of Raman spectra of Aβ oligomers and its solvents; transmitted light image of AD model cells; comparison of lipid components between Aβ and control groups; Raman spectra of Aβ oligomers and Aβ fibrils; cytochrome Raman intensity of AD model cells; Alamar blue assay of AD model cells; immunofluorescence images of cytochrome *c* and COX4 in AD model cells; confirmation of reduced Aβ concentration in Aβ solution following immunodepletion with Lecanemab; immunofluorescence images of cytochrome *c* and COX4 in Lecanemab treated AD model cells; primary antibodies, secondary antibodies, and primers used in this research; apparent size differences between cytochrome Raman and immunofluorescence images (PDF)
(PDF)

## Abbreviation

BDNF: brain derived neurotrophic factor
