# Supplementary material for: Label-Free Detection of Cellular Aβ Accumulation and Mitochondrial Dysfunction in AD Cell Models via Raman Microscopy
Source: Anal Chem. 2026 Jun 23;98(26):19405–17. doi: 10.1021/acs.analchem.5c06200 (PMC13348670; doi:10.1021/acs.analchem.5c06200)
Supplement: Supplementary file 1 [file ac5c06200_si_001.pdf]

Supporting Information

Label-free detection of cellular Aβ accumulation and mitochondrial dysfunction in AD cell models via Raman microscopy

Yusuke Mitsuoka[1], *Takeshi Morimoto*[1,2]*, *Kazuki Bando*[3,4], *Susumu Hara*[1,5,6], *Katsumasa Fujita*[3,4,7], *Kohji Nishida*[1,5,8]

Affiliations

[1]Department of Ophthalmology, The University of Osaka Graduate School of Medicine, Suita, Osaka 565-0871, Japan.

[2]Department of Advanced Visual Neuroscience, The University of Osaka Graduate School of Medicine, Suita, Osaka 565-0871, Japan.

[3]Department of Applied Physics, Osaka University, Suita, Osaka 565-0871, Japan.

[4]Advanced Photonics and Biosensing Open Innovation Laboratory, National Institute of Advanced Industrial Science and Technology (AIST), Suita, Osaka 565-0871, Japan.

[5]Premium Research Institute for Human Metaverse Medicine (WPI-PRIMe), The University of Osaka, Suita, Osaka 565-0871, Japan.

[6]Transdimensional Life Imaging Division, Institute for Open and Transdisciplinary Research Initiatives, The University of Osaka, Suita, Osaka 565-0871, Japan.

[7]Institute for Open and Transdisciplinary Research Initiatives, Osaka University, Suita, Osaka 565-0871, Japan.

[8]Integrated Frontier Research for Medical Science Division, Institute for Open and Transdisciplinary Research Initiatives, Osaka University, Suita, Osaka 565-0871, Japan.

*Correspondence to: Takeshi Morimoto

Email: takeshi.morimoto@ophthal.med.osaka-u.ac.jp

Table of Contents

Figure S1. Neuron-differentiated SH-SY5Y cells exhibit neuronal properties…………..…S3
Figure S2. Analysis methods of Raman spectra…………..…………………………………S4

Figure S3. Comparison of Raman spectra of Aβ oligomers, HEPES, and DMSO…………S6

Figure S4. Transmitted light image of AD model cells……………………………………...S7

Figure S5. Comparison of lipid components between Aβ and control groups………………S8

Figure S6. Raman spectra of Aβ oligomers and Aβ fibrils…………………………………S9

Figure S7. Cytochrome Raman intensity of AD model cells……………………….…..S10
Figure S8. Alamar Blue assay results for AD model cells…………………………………S11
Figure S9. Immunofluorescence images of cytochrome c and COX4 in AD model cells…..S12

Figure S10. Aβ solution concentration after immunodepletion……………………….………S13

Figure S11. Immunofluorescence images showing cytochrome c and COX4 in cells treated with Lecanemab or control IgG…………………………………………..……………S14
Table S1. The primary antibodies used in this study. ……………………………………S15
Table S2. The secondary antibodies used in this study. ……………………………..………S16
Table S3. The primers used for real-time RT-PCR in this study. ………………………..……S17
Supplementary Note 1. Apparent size differences between cytochrome Raman and immunofluorescence images………………………………………...………………..S18

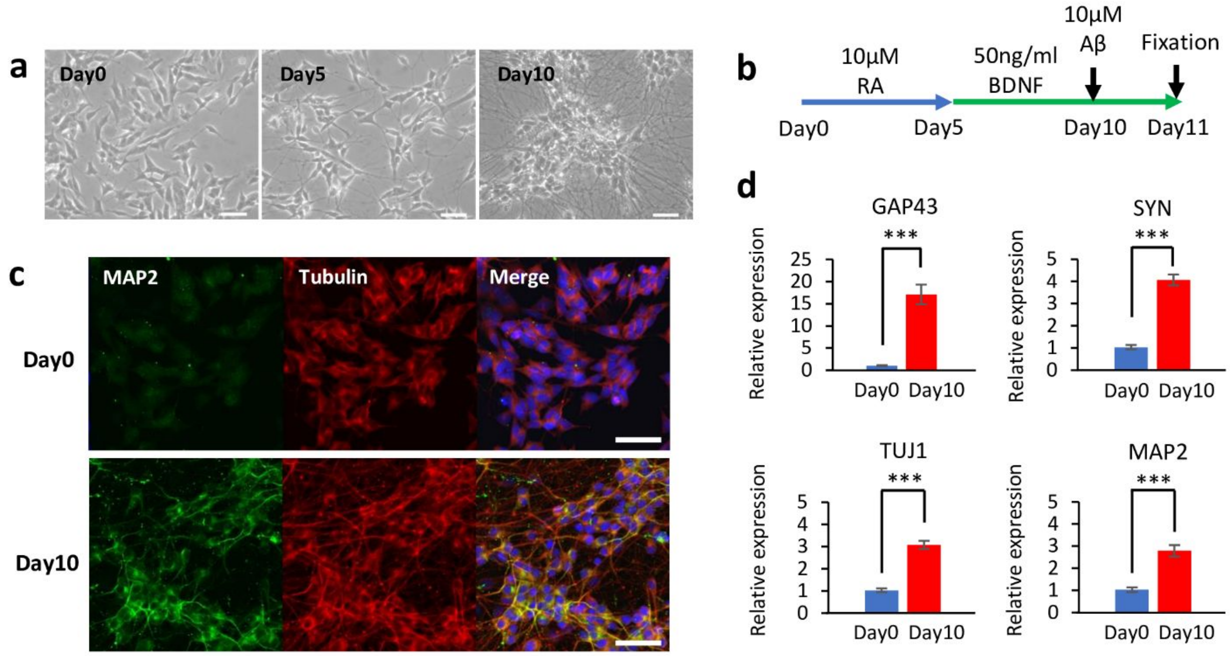

Figure S1. Neuron-differentiated SH-SY5Y cells exhibit neuronal properties.

(a) Transmitted-light images of the differentiation process. By day 10, differentiated cells extend numerous neurites. Scale bar: 50 μm.

(b) Schematic of the neural differentiation protocol. Cells were treated with 10 μM RA for 5 days, followed by 50 ng/mL BDNF for 5 days. After differentiation, cells were exposed to 10 μM Aβ oligomers for 24 h, fixed, and analyzed using Raman microscopy.

(c) Immunofluorescence images of undifferentiated and differentiated (day 10) SH-SY5Y cells. Differentiated cells show strong MAP2 staining, indicative of neuronal identity. Scale bar: 50 μm.

(d) RT-PCR analysis of neural differentiation marker expression. All markers showed higher expression levels on day 10 compared to day 0 (N = 7 each). Gene expression levels of day 10 cells are shown relative to day 0 (set to 1). ***$P < 0.001$, t-test.

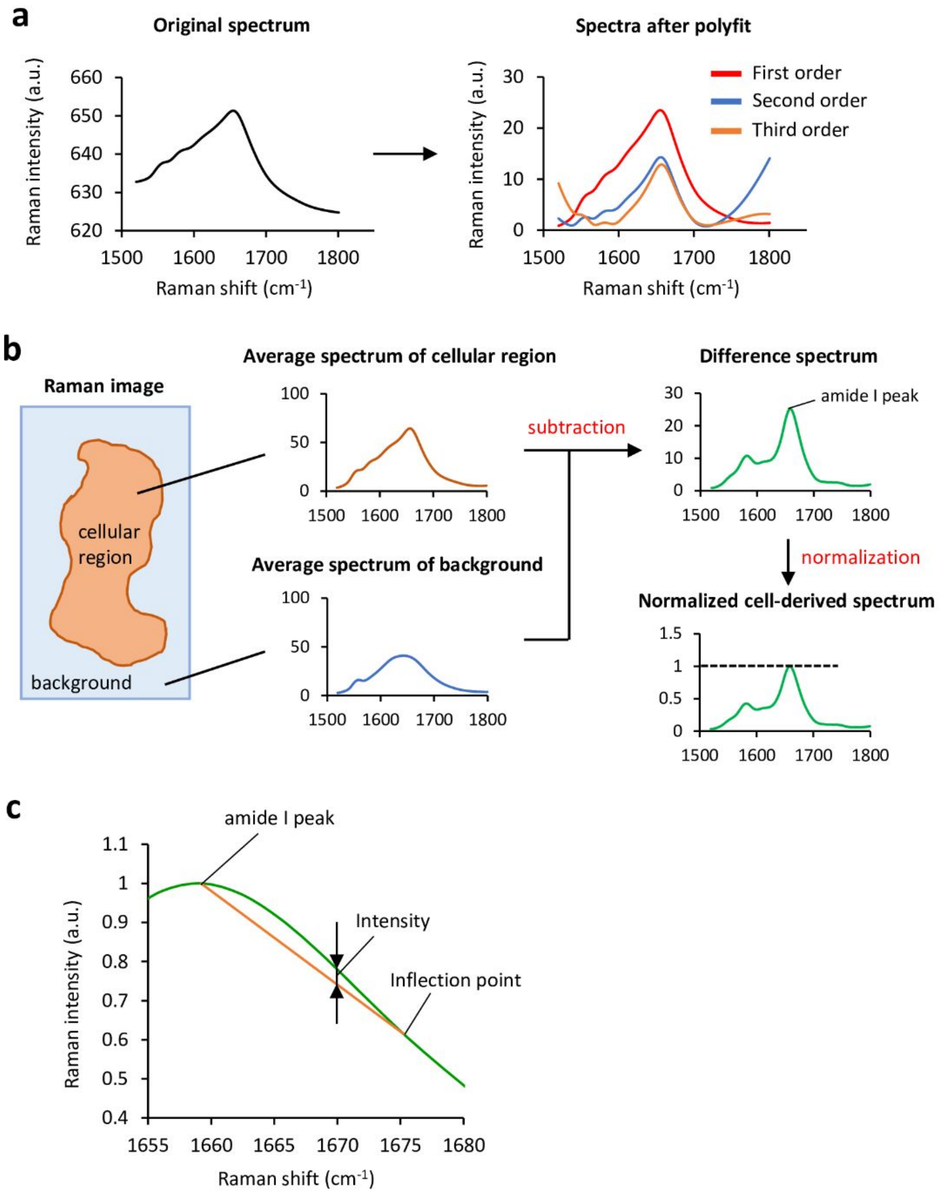

Figure S2. Analysis Methods of Raman Spectra.

(a) Example of polynomial fitting of different orders applied to the amide I region of a cellular spectrum. For all orders other than the first, the fitted curves exhibited oscillations. Since the first-order polyfit best preserved the shape of the original spectrum and provided a stable baseline, we adopted the first-order polyfit in this study.

(b) Spectral processing method for the amide I region. The spatially averaged spectrum from the intracellular region and that from the extracellular region were each measured, and a difference spectrum was obtained by subtracting the latter from the former. The spectrum was then normalized by dividing it by the maximum intensity of the amide I band.

(c) Quantification method for Aβ Raman intensity. A line segment was drawn between the amide I peak and the inflection point on the right side of the peak. The Raman intensity was defined as the vertical distance between the spectral value at 1670 cm^{-1} and this line segment. The inflection point was determined by calculating the second derivative of the Raman spectrum and identifying the point where the second derivative crossed zero with a sign change.

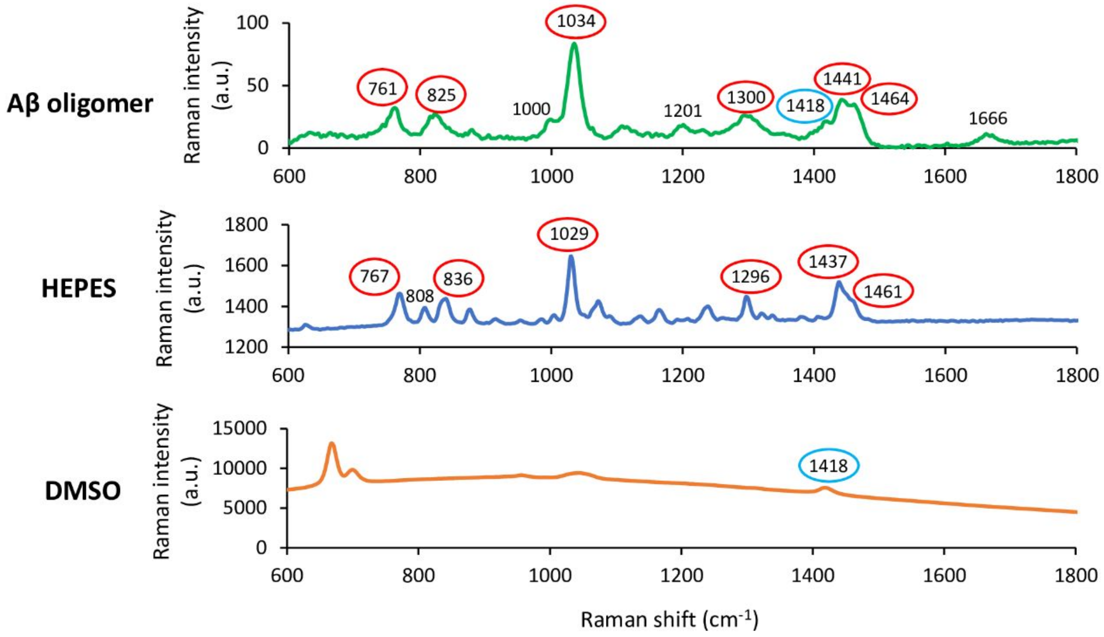

Figure S3. Comparison of Raman spectra of Aβ oligomers, HEPES, and DMSO.

The Raman spectrum of Aβ oligomers shown here is identical to that presented in Figure 1. In addition to the peak at 1666 cm^{-1}, several additional peaks are observed; however, these peaks correspond closely to those found in the Raman spectra of HEPES and DMSO, the solvents used for preparing the Aβ oligomers. Peaks shared between Aβ oligomers and HEPES are circled in red, whereas those shared between Aβ oligomers and DMSO are circled in blue. These observations indicate that most peaks other than the one at 1666 cm^{-1} are attributable to the solvents rather than to Aβ oligomer–specific vibrational modes.

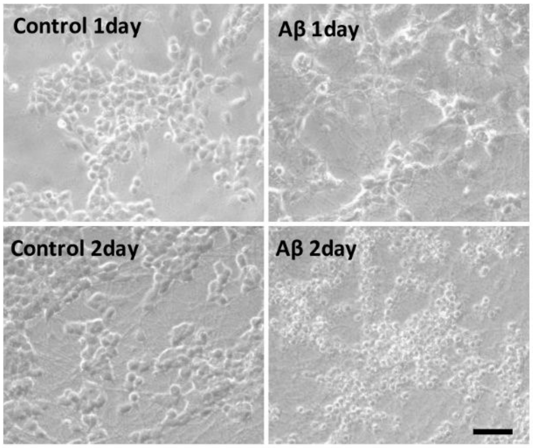

Figure S4. Transmitted light image of AD model cells.

Representative transmitted light images of cells treated with Aβ or vehicle (control). In the Aβ group, abnormalities in cell morphology and neurite structure were observed one day after Aβ administration, and most cells underwent cell death by day two. In contrast, no morphological changes were observed in the control group. Scale bar: 50 μm.

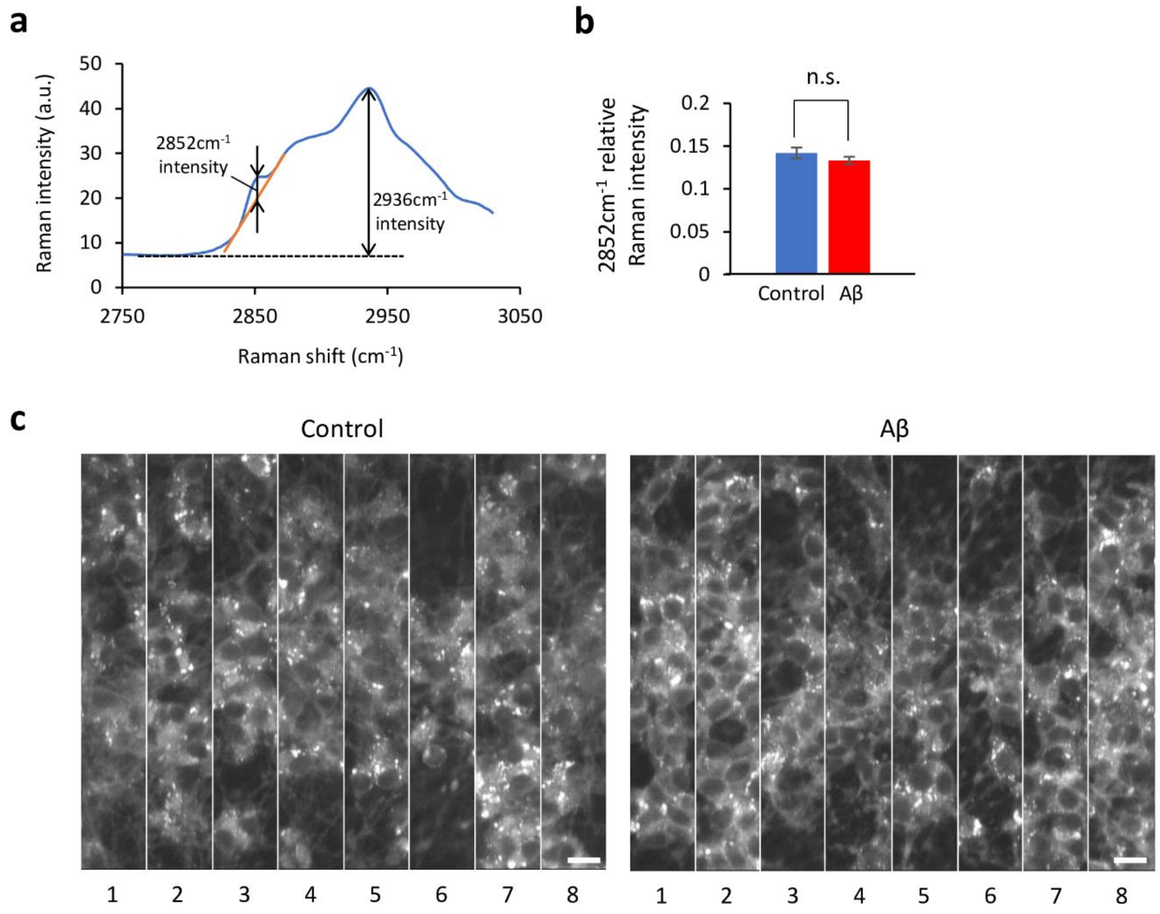

Figure S5. Comparison of lipid components between Aβ and control groups.

(a) Method for measuring the relative Raman intensity at 2852 cm^{-1}. The Raman intensity at 2852 cm^{-1} (lipid CH_2) was measured by drawing a tangent to the spectrum and defining the distance between the curve and the tangent as the Raman intensity. In contrast, the distance from the baseline to the peak at 2936 cm^{-1} was defined as the Raman intensity of the protein and lipid CH_3/CH_2 stretching. The Raman intensity at 2852 cm^{-1} was then normalized to the intensity at 2936 cm^{-1}.

(b) A comparison of the Raman intensity at 2852 cm^{-1} within the cellular regions between the Aβ group and the control group showed no statistically significant difference. Control: N = 29 images (Sample 1: 8, Sample 2: 8, Sample 3: 7, Sample 4: 6); Aβ: N = 29 images (Sample 1: 8, Sample 2: 8, Sample 3: 6, Sample 4: 7). t-test.

(c) Raman images at 2852 cm^{-1}. For both the Aβ and control groups, Raman images from eight locations within a single sample measured under identical conditions on the same day are shown. No apparent changes in lipid distribution were observed between the two groups. Scale bar: 20 μm.

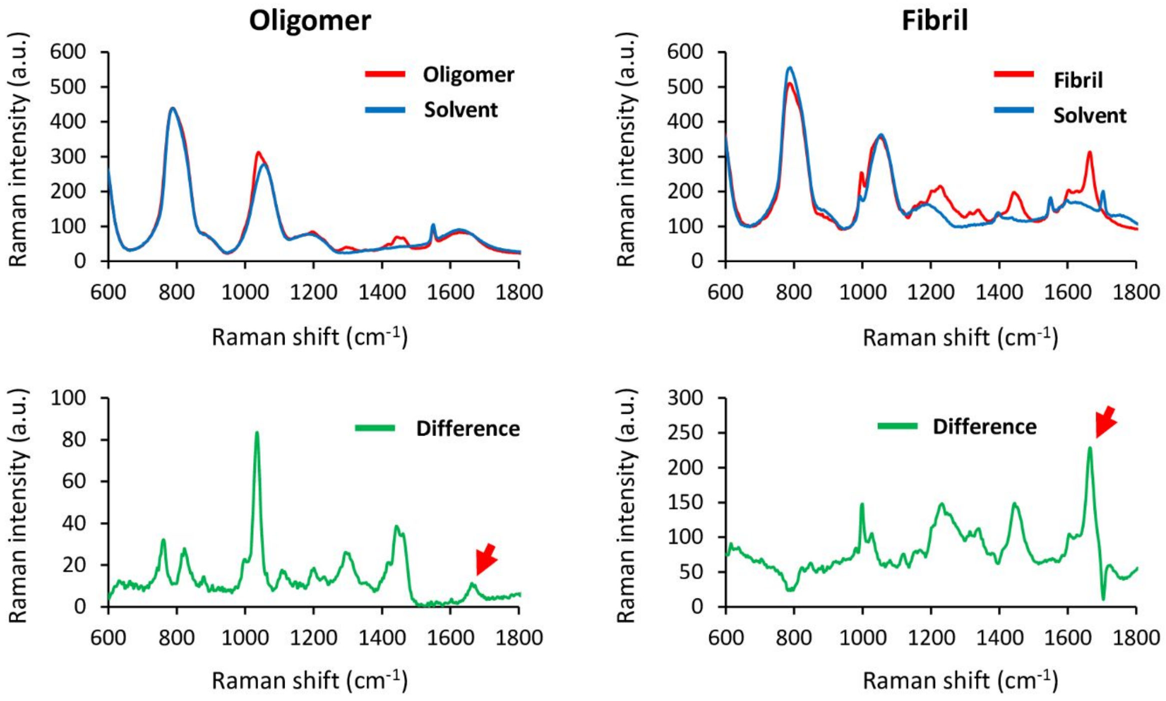

Figure S6. Raman spectra of Aβ oligomers and Aβ fibrils.

Aβ oligomer and fibril solutions were dropped onto quartz glass and air-dried prior to Raman measurement. Raman spectra of the solvent alone were also measured, and solvent-subtracted spectra were calculated. The peak at 1666 cm^{-1}, indicative of the β-sheet structure of Aβ, is marked with a red arrow. The intensity of this peak was higher in Aβ fibrils than in Aβ oligomers.

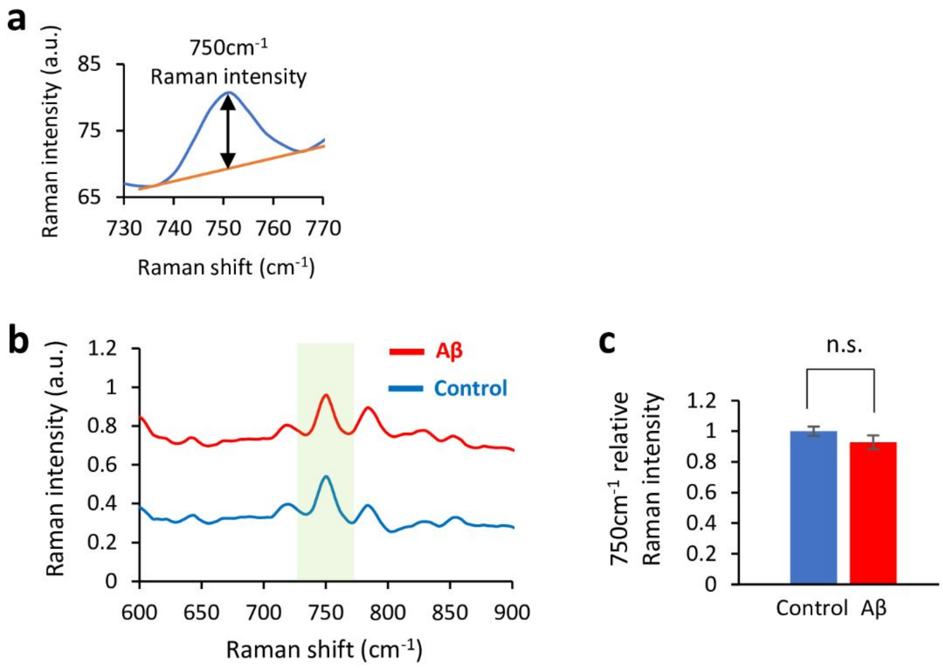

Figure S7. Cytochrome Raman intensity of AD model cells.

(a) Definition of the Raman intensity at 750 cm^{-1}. A straight line was drawn tangent to the curve, and the vertical distance between the curve and the straight line at 750 cm^{-1} was defined as the Raman intensity. The intensity was normalized to the amide I peak.

(b) Raman spectra in the region near 750 cm^{-1}. The spectra of the Aβ and control groups

represent the average intracellular spectra obtained from eight Raman images acquired at different locations within each sample.

(c) Quantification of Raman intensity at 750 cm^{-1} using the method described in (a). No significant difference was observed between groups. Control: N = 29 images (Sample 1: 8,

Sample 2: 8, Sample 3: 7, Sample 4: 6); Aβ: N = 29 images (Sample 1: 8, Sample 2: 8, Sample

3: 6, Sample 4: 7). t-test.

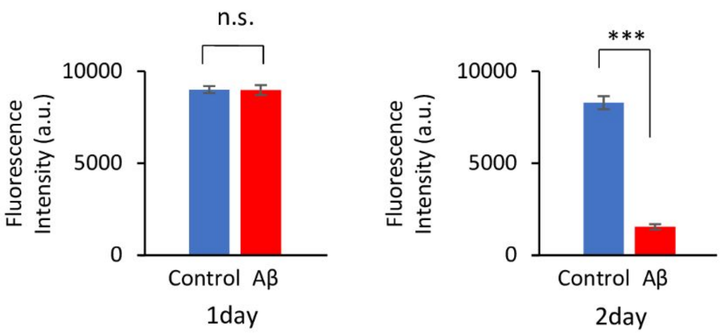

Figure S8. Alamar Blue assay results for AD model cells.

No significant difference was observed between the control and Aβ groups one day after Aβ administration. However, a decrease in fluorescence intensity was detected in the Aβ group two days after administration, indicating mitochondrial dysfunction. N = 4 samples. ***$P < 0.001$, t-test.

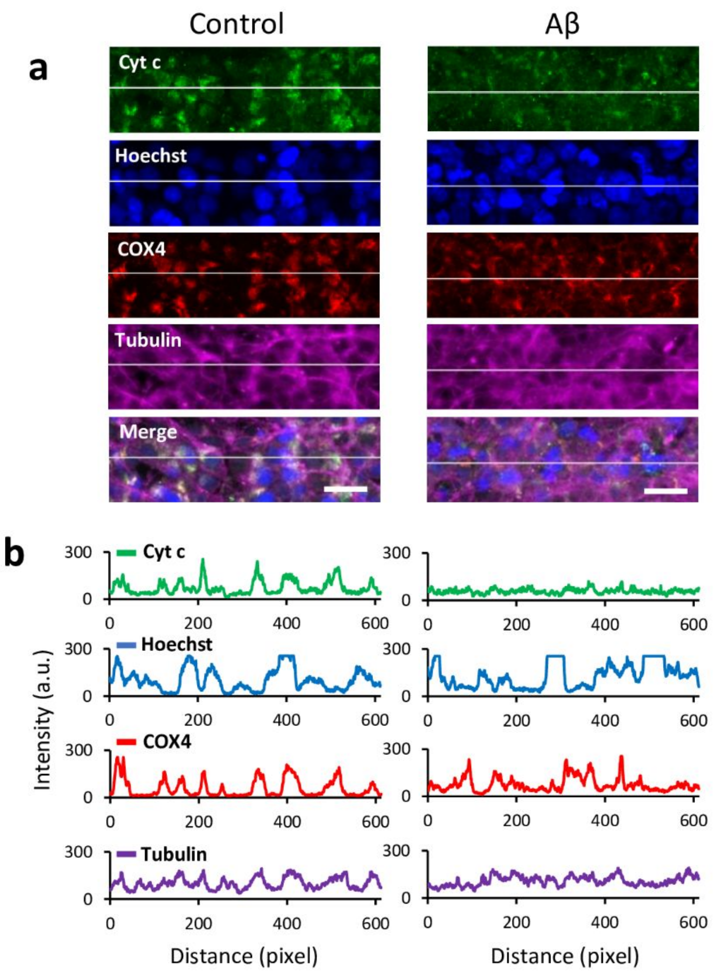

Figure S9. Immunofluorescence images of cytochrome c and COX4 in AD model cells.
(a) Immunofluorescence staining of cytochrome c and COX4. (b) Plot profiles corresponding to panel (a). In the control group, cytochrome c colocalized with mitochondria labeled with COX4, displaying a punctate pattern. Accordingly, the plot profiles of cytochrome c and COX4 showed similar waveforms. In contrast, in the Aβ group, mitochondria indicated by COX4 were fragmented and diffusely distributed throughout the cells. The plot profiles of cytochrome c and COX4 did not overlap, suggesting leakage of cytochrome c from mitochondria. Scale bar: 20 μm.

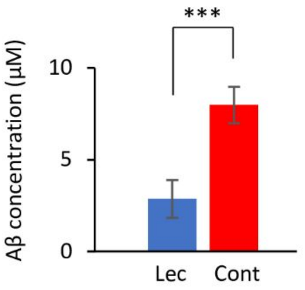

Figure S10. Aβ solution concentration after immunodepletion.

Aβ concentrations in solutions immunodepleted with either Lecanemab or control IgG were quantified by ELISA. Lecanemab treatment significantly reduced Aβ levels compared with control. Data are presented as mean ± SD (N = 3). ***$P < 0.001$, t-test.

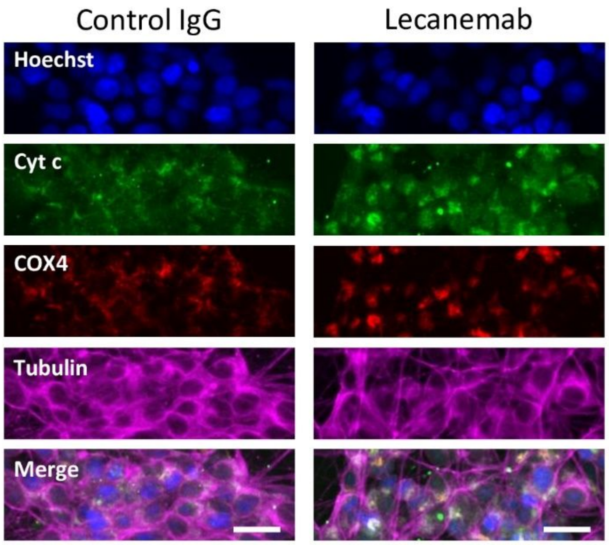

Figure S11. Immunofluorescence images showing cytochrome c and COX4 in cells treated with Lecanemab or control IgG.

In the control IgG group, mitochondria labeled by COX4 appeared fragmented and dispersed throughout the cells. In contrast, in the Lecanemab group, cytochrome c colocalized with COX4-positive mitochondria in a punctate pattern. Scale bar: 20 μm.

Table S1. The primary antibodies used in this study.

Antibody	Type	Company	Catalog number	Dilution
Anti-Human Amyloidβ E22P (11A1)	Mouse	Immuno-Biological Laboratories	10379	1:100
Anti-Beta III Tubulin Antibody	Chicken	Sigma-Aldrich	AB9354	1:100
anti-cytochrome C antibody	Rabbit	abcam	ab133504	1:100
anti-COX IV antibody	Mouse	abcam	ab33985	1:1000
Anti-MAP2 antibody	Rabbit	abcam	ab32454	1:1000

Table S2. The secondary antibodies used in this study.

Antigen	Type	Fluorophore	Company	Catalog number	Dilution
Mouse IgG	Donkey	Alexa Fluor 488	Invitrogen	A21202	1:500
Mouse IgG	Donkey	Alexa Fluor 568	Invitrogen	A10037	1:500
Rabbit IgG	Donkey	Alexa Fluor 488	Invitrogen	A21206	1:500
Rabbit IgG	Donkey	Alexa Fluor 568	Invitrogen	A10042	1:500
Chicken IgY	Goat	Alexa Fluor 647	abcam	ab150171	1:500

Table S3. The primers used for real-time RT-PCR in this study.

Gene	Forward (5'→3')	Reverse (5'→3')
GAP43	GAGCAGCCAAGCTGAAGAGAAC	GCCATTTCTTAGAGTTCAGGCATG
SYN	TCGGCTTTGTGAAGGTGCTGCA	TCACTCTCGGTCTTGTTGGCAC
MAP2	TGCGCTGATTCTTCAGCTTG	TGTGTCGTGTTCTCAAAGGGT
TUJ1	GGCCAAGGGTCACTACACG	GCAGTCGCAGTTTTCACACTC
GAPDH	TCGTGGAAGGACTCATGACC	AGGCAGGGATGATGTTCTGG

Supplementary Note 1.
Apparent size differences between cytochrome Raman and immunofluorescence images.

In the Raman images of cytochromes, the cytochrome-positive regions appeared slightly larger than those observed in the corresponding immunofluorescence images. In this section, we discuss the possible causes of this discrepancy.

First, there are fundamental optical differences between the Raman imaging system and the confocal microscope used for immunostaining, which are considered to be the primary contributors to the observed discrepancy. Raman imaging was performed using a 25× objective with a numerical aperture (NA) of 1.1 in a slit-confocal configuration, whereas immunofluorescence images were acquired using a 20× objective with an NA of 0.7 in a point-confocal system. In slit-confocal Raman microscopy, the confocal effect is present only along one lateral axis, while the orthogonal axis exhibits wide-field–like characteristics. This optical configuration increases sensitivity to defocus, thereby making object boundaries appear expanded.

Differences in spatial sampling further contribute to this effect and are also regarded as a major factor. Immunofluorescence images were acquired with a spatial resolution of 311 nm per pixel, whereas Raman images were obtained with a scan step size of 635 nm to cover a larger field of view and to minimize local heat generation during acquisition. This coarser sampling in Raman imaging leads to spatial smoothing of image features, making object contours appear broader compared with immunostaining images. In addition, the inherently lower signal-to-noise ratio of Raman signals further enlarges the apparent size of positive regions.

Beyond these imaging-related factors, a key biological difference lies in the cytochrome species detected by each method. The Raman signal around ~750 cm^{-1} reflects contributions from multiple cytochrome species in various redox states, whereas immunostaining specifically detects cytochrome c. Furthermore, the immunostaining procedure itself may influence cytochrome distribution, as permeabilization, antibody incubation, and other processing steps can induce physiological changes such as alterations in redox balance or protein localization.

Importantly, quantitative analysis based on the standard deviation of image intensity revealed no significant differences between Raman and immunofluorescence measurements. Therefore, although the apparent sizes of cytochrome-positive regions differ between the two imaging modalities, these differences do not affect the quantitative assessment of cytochrome distribution.
